# Caring for terminally Ill patients: the impact on oncologists

**DOI:** 10.1186/s12904-024-01562-9

**Published:** 2024-09-28

**Authors:** Nagavalli Somasundaram, Halah Ibrahim, Ranitha Govindasamy, Nur Amira Binte Abdul Hamid, Simon Yew Kuang Ong, Lalit Kumar Radha Krishna

**Affiliations:** 1 Division of Cancer Education, National Cancer Centre Singapore, 30 Hospital Boulevard, Singapore, 168583 Singapore; 2 Duke-NUS Medical School, National University of Singapore, 8 College Road, Singapore, 169857 Singapore; 3 Division of Medical Oncology, National Cancer Centre Singapore, 30 Hospital Boulevard, Singapore, 168583 Singapore; 4 Department of Medical Sciences, Khalifa University College of Medicine and Health Sciences, Abu Dhabi, United Arab Emirates; 5 Division of Palliative and Supportive Care, National Cancer Centre Singapore, 30 Hospital Boulevard, Singapore, 168583 Singapore; 6 Yong Loo Lin School of Medicine, National University of Singapore, NUHS Tower Block, Level 11, Block 1E, Kent Ridge Road, Singapore, 119228 Singapore; 7 Centre for Biomedical Ethics, National University of Singapore, Block MD11, 10 Medical Drive #02-03, Singapore, 117597 Singapore; 8 End of Life Care Centre, Cancer Research Centre, Palliative Care Institute Liverpool, University of Liverpool, Academic Palliative &200 London Road, Liverpool , Liverpool, L3 9TA UK; 9 PalC, The Palliative Care Centre for Excellence in Research and Education, Dover Park Hospice, 10 Jalan Tan Tock Seng, Singapore, 308436 Singapore; 10 Health Data Science, University of Liverpool, Whelan Building, The Quadrangle, Brownlow Hill, Liverpool, Liverpool, L69 3GB UK

**Keywords:** Oncology, Death, Dying, Doctor-patient relationship, Professional identity formation, Palliative care, Professionalism

## Abstract

**Background:**

Journeying with patients throughout their cancer trajectory and caring for them at the end of life can lead to emotional and moral distress in oncologists, negatively impacting their personal and professional identities. A better understanding of how transitions in care goals affect oncologists can shed light on the challenges faced and the support required. This study explored the impact of care transitions on oncologists’ professional identity formation (PIF).

**Methods:**

From September to December 2023, semi-structured interviews were conducted with oncologists in a palliative care center in Singapore. The Ring Theory of Personhood (RToP) was used as a framework to capture the effects of experiences with patients transitioning from curative to palliative care on the oncologists’ sense of self and identity. Data were analyzed using both inductive and deductive qualitative analysis.

**Results:**

Participants included six female and six male physicians, aged 30 to 53 years (mean 38 years), with an average of 9.75 years of experience as oncologists. The main domains identified were 1) challenges faced in transitioning patients to palliative care, 2) the impact of dealing with dying patients on oncologists, and 3) coping mechanisms.

**Conclusion:**

Oncologists experience self-doubt and moral distress as they manage transitions in care. The PIF of oncologists can be supported through reflection and introspection, peer support, and interventions to promote self-care — ultimately enabling them to make meaning of their experiences, renew family ties, and reaffirm their commitment to the profession.

**Supplementary Information:**

The online version contains supplementary material available at 10.1186/s12904-024-01562-9.

## Background

Numerous studies have explored the impact of patient death on physicians [1–4]. Emotional exhaustion, detachment, and depersonalization are well-documented, while long-term concerns include absenteeism, poor interprofessional working relationships, and suboptimal patient care [1–5]. This ‘cost of caring’ affects physicians personally and professionally and can impact their professional identity formation (PIF). PIF is often conceptualized as a socialization process transforming an individual from layperson to physician [6]. It involves internalizing and manifesting a profession’s core values and beliefs [7]. Yet, it is inextricably linked to personal identity or personhood [8]. While fundamental elements of personhood and identity remain foundational and enduring, physicians undergo a myriad of social, personal, academic, professional, and clinical experiences that shape their professional identity — often in non-linear or unpredictable ways. This is especially evident in oncology, where daily interactions can be distressful and emotion-laden, leaving indelible marks on those involved.

Sinclair [9] noted that among palliative care nurses and physicians, acknowledging the inevitability of death and focusing on maximizing comfort and dignity helped them to find meaning in their work and nurtured their PIF, thereby affirming their self-concepts of identity and personhood [10, 11]. For many oncologists, however, repeated and prolonged exposure to dying patients and their grieving families can evoke feelings of guilt [12], helplessness [12], failure [13], grief [13, 14], and depression. Granek et al. [15] reported that oncologists undergo a unique mourning experience resulting in sadness, sleep loss, and difficulties in maintaining emotional boundaries, which can lead to emotional distancing from dying patients. Moreover, this grief can negatively impact relationships with colleagues, family, and friends. Though these effects are well-recognized, personalized support for health professionals has been slow and often inadequate [10]. Studies have emphasized the need for individualized and context-specific interventions to better support health professionals in dealing with dying patients [11, 16–19]. To date, research on oncologists’ grief has primarily focused on burnout and subsequent lapses in patient care. There is limited understanding of how caring for dying patients affects the PIF of oncologists. Amidst reports of increasing burnout, dissatisfaction, and attrition, it is important to identify factors that support or threaten the professional identity of oncologists.

### The Ring Theory of Personhood

Current frameworks do not fully consider the dynamic influence of physicians’ changing beliefs, values, and attitudes on their behavior and decision-making [20, 21]. To explore these gaps, we use Krishna and Alsuwaigh’s [22] Ring Theory of Personhood (RToP) to guide our research question:* “How does caring for patients transitioning from curative to palliative treatment impact the professional identity of oncologists?”* The RToP considers the evolving nature of personhood and the different sources that influence and inform one’s self-concept of identity. The model helps to frame the effects of caring for the dying on a physician’s personhood and maps changes in belief systems and the impact of these changes on the physician’s conduct, coping, and well-being. The RToP posits that self-concepts of personhood revolve around four domains: innate, individual, relational, and societal. Several studies have proposed that a better understanding of the shifts in the belief systems contained within the four domains of personhood can help tailor the support that individuals require [17, 18, 23, 24].

**Fig. 1:**
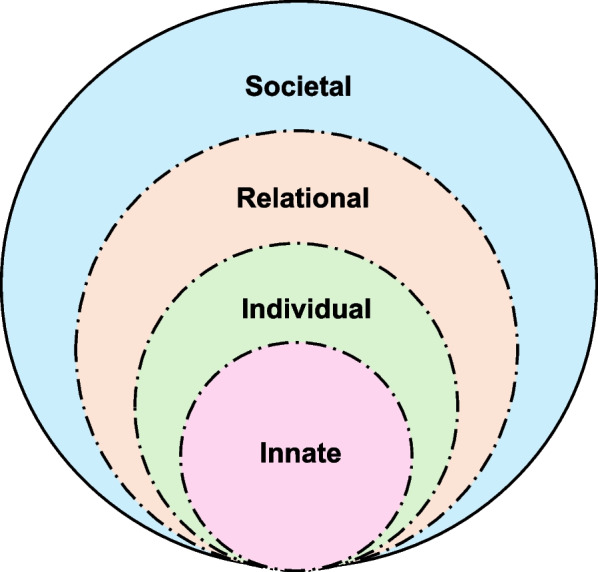
The RToP rings [27]

At the center of the RToP is the innate ring, which includes genetic and cultural influences, such as gender and ethnicity, that shape the individual from birth [22]. The individual ring encompasses an individual’s values and beliefs, manifesting through their ability to communicate and display emotions [25]. For example, a devout individual’s thoughts, beliefs, and actions can reflect their religious stance. The relational ring comprises important personal relationships, and the societal ring consists of peripheral relationships, such as those with colleagues [26] (See Fig. 1).

Chan and Chia [24] and Kuek and Ngiam [17] introduce the concepts of *resonance, synchrony, disharmony,* and *dyssynchrony* to characterize conflicts within and between the four rings. *Resonance* is present when environmental and professional influences are consistent with the physician’s belief systems. Synchrony occurs when these resonant belief systems are reprioritized to better fit with practical considerations. Disharmony arises when the clinician’s beliefs, values, and principles conflict with those introduced within one of the rings – for instance, a clinician’s professional expectations could conflict with work obligations*. Dyssynchrony* occurs with conflicts between the rings, such as when clinicians’ professional expectations (societal ring) interfere with their personal lives (individual ring). Further examples of these concepts can be found under Domain 1 of the ‘Results’ section. Adaptations made to a physician’s belief systems to address *disharmony* and *dyssynchrony* can lead to moral distress and its sequelae [22].

## Methods

The research team used Krishna’s Systematic Evidence-Based Approach (SEBA) [10, 11, 28–31] to conduct qualitative interviews. This qualitative study aims to characterize how caring for patients transitioning from curative treatment to palliative care impacts the professional identity of oncologists. Ethics approval (2021/2176) was obtained from the SingHealth Combined Institutional Review Board.

First, an expert team consisting of a medical librarian, clinicians, and clinician-educators from an oncology center, palliative care institute, and local medical schools worked with a research team of clinicians and medical trainees to develop a semi-structured interview questionnaire. The interview questions aimed to elicit the impact of the lived experiences of oncologists on their personal beliefs, values, principles, and practices [32]. Through a modified Delphi process [33], local palliative care and oncology physicians and qualitative researchers then reviewed and revised the interview guide (Additional File 1).

### Data collection

In September 2023, all oncologists at a tertiary care cancer center in Singapore received email invitations to participate in the study. Emails included participant information sheets, study information, and details on the study's nature, duration, and aims. The invitations emphasized participant anonymity and the participant’s right to withdraw without prejudice at any point. Participation was voluntary, and no incentives were offered. Verbal and written consent was obtained before conducting the interviews. Between 12^th^ September 2023 and 17^th^ December 2023, two trained research team members conducted one-on-one interviews on an institutionally secured Zoom video-conferencing platform. Each interview was conducted in English, audio-recorded with permission, and lasted approximately 45 min. Data collection and analysis occurred concurrently. When two consecutive interviews did not yield new themes or insights, the research and expert teams concluded recruitment and no new interviews were conducted. Audio recordings were transcribed verbatim, anonymized, and sent to each participant for member checking to confirm that the transcripts accurately conveyed their perceptions.

### Data analysis

Data analysis was conducted concurrently using Hsieh and Shannon’s [34] approach to directed content analysis and Braun and Clarke’s [35] thematic analysis. This concurrent analysis enabled a review of the data from different perspectives.

Two independent teams of at least two authors carried out thematic and content analyses. To ensure rigor and trustworthiness, the team members independently analyzed the data- manually coding, identifying subthemes from the codes, creating themes from subthemes, sharing broader patterns of meaning, and maintaining an audit trail. The categories for the direct content analysis were drawn from Kuek and Ngiam’s [17] systematic scoping review entitled “*The impact of caring for dying patients in intensive care units on a physician's personhood: a systematic scoping review*” and Ho et al.’s [10] study entitled “*Physician–patient boundaries in palliative care*.” Sandelowski and Barroso’s approach to “negotiated consensual validation” was used to reach a consensus between the teammates [36]. Finally, overlapping themes and categories were identified using Phases 4 to 6 of France et al.’s [37] adaptation of Noblit and Hare’s [38] seven phases of meta-ethnography [37]. Reciprocal translation was employed to determine if the themes and categories can be used interchangeably. The first (NS), second (HI), and last authors (LKRK) reviewed all the codes, subthemes, and themes as part of the iterative process. The final themes were discussed among all of the authors. The combined themes and categories are referred to as domains.

## Results

There were six female and six male participants, all practicing physicians aged between 30 and 53 years (mean 38 years), with an average of 9.75 (range 4–12) years of experience as oncologists. The main domains identified are 1) challenges faced in transitioning patients to palliative care, 2) the impact of dealing with dying patients on oncologists, and 3) coping mechanisms. The domains are framed within the RtoP, and supporting quotes are included.

### Domain 1: challenges faced in transitioning patients to palliative care

Oncologists faced many challenges when deciding to transition their patients to palliative treatment, including disagreements within the healthcare team and resistance from patients and their families. Moreover, some oncologists focused on curative treatment and did not consider psychosocial care and communication about end-of-life care to be within their scope of service.

#### Disharmony in the societal ring

The decision to discontinue curative treatment weighed heavily on some oncologists. They often grappled with justifying their decisions to stop treatment, viewing it as an admission of failure.

You’re a consultant; the responsibility lies with you, so you have to think very carefully. Once you stop [curative treatment], there’s no turning back. (Dr 5)

I do feel conflicted about treatment decisions a lot and it's not the most pleasant thing in the world… to feel like you have to make certain decisions that… determines outcomes for the patient. And you may be wrong. (Dr 8)

At times, oncologists felt pressured by other members of the healthcare team to continue aggressive treatment that they believed to be futile.

When you feel like the patient has had many lines of treatment and they're really not fit for any more treatment, but team members want to continue when the patient themselves has said they don't want anymore, I think that can be quite troubling… [Or] the patient and the doctor know that treatment is not going to work for the patient, but they insist on having more. I think that is also a dilemma. (Dr. 11)

Most oncologists believe in having honest conversations about illness and treatment. However, this was sometimes challenged by non-accepting patients and families who resisted the transparent disclosure of diagnostic or prognostic information. As a result, the physicians experienced feelings of guilt and helplessness.

Sometimes, I feel a bit helpless when you are not able to get through to the patient or the family [who]… couldn’t let go of a lot of treatment decisions. Sometimes, you feel there’s only so much you can say to somebody to explain things, and reasoning doesn’t really work with these patients anyway. (Dr 2)

This part of being honest and telling patients… but at the same time, you have family members who would say, “Don’t tell them; they won’t be able to take it.” So, there are times when you feel you agree with them, but I feel conflicted because if it was me, I would want to know. (Dr2)

#### Dyssynchrony between societal and relational rings

Caring for ill and dying patients fostered close, sometimes intimate relationships with patients, which was, at times, a source of stress for oncologists who struggled to navigate blurred relationship boundaries.

I was so worried that [the relationship] was unprofessional… I just cannot think about this imaginary line that we are drawing upon ourselves. Where is it that we should keep that line off in terms of relationships with patients… Because this is what I'm taught in medical school. Medical school tells me, “Don’t have this physician transference.” Whenever we talk about that, we always split it [separate the professional and emotional aspects of the physician-patient relationship]. But I just don't feel that it should be split now that I'm a bit older. (Dr 6)

#### Dyssynchrony between societal and individual rings

Many oncologists struggled with work-life balance and felt that their commitment to their patients and duties often sacrificed family time or personal well-being.

It was draining. It was mentally and physically draining. If I go on vacation, my phone is on all the time because I really am texting the teams. I feel compelled. I’m on vacation, and I still feel compelled to answer them. (Dr. 4)

### Domain 2: the impact of dealing with dying patients on oncologists

Caring for patients in the transition to palliative and end-of-life care often caused distress and, for some of the oncologists, led to disengagement and burnout. These experiences made them doubt their clinical skills and decision-making abilities and question their career choices. Despite the challenges, many oncologists found these transitions meaningful, reaffirming their spirituality and personal relationships. Several reflected on how their role as oncologists empowered them to help their patients and to form closer bonds with peers and loved ones.

#### Negative impact on societal ring

The decision to transition to palliative care brought on complex emotions, including helplessness, guilt, and self-doubt. These feelings threatened the physicians’ confidence and raised questions about their competence, regardless of experience or years of practice. The death of patients with whom oncologists had developed personal relationships resulted in a profound loss and distress that “takes a part of you away” (Dr. 3). Some of these experiences were so traumatic that the oncologists questioned the future of their careers. For others, the emotionally taxing and distressing nature of the profession numbed their ability to connect with or show compassion for their patients.

The team was very distressed, and we had to have a debrief after that… I found it very difficult to accept it [the death]. We actually went back to do a post-mortem of the situation, and I attended her wake as well. And I remember going off from the wake crying very terribly. (Dr 3)

I signed the Do Not Resuscitate order for him. And I think one day later, he had passed… It was quite bad. I think that was the very first time that I actually thought I wanted to quit. I remember walking around the whole hospital compound, looking for somebody familiar that I could talk to, but it was already 7/8 pm at night. So, there wasn't really anyone around. (Dr 3)

I didn’t have any spare emotion to give him… I was an empty vessel at the time. My role in the day was to just get through the day and deal with his medical issues as I see it. And I just didn’t have that compassion, in a way, in me left to give, to allot him a bit more time because I just couldn’t build a rapport. (Dr 4)

#### Negative impact on innate ring

Repeatedly witnessing the suffering of patients caused oncologists to ask existential questions about spirituality, religion, life, and death.

Once you have a cancer diagnosis, you have a lot of questions about what it means to have a meaningful life. Questions about fate and what does God have in store for you? I mean, the Christian worldview is that life on Earth is just a prelude to the afterlife and meeting God. But I think being in oncology has definitely forced me to think about this a lot more. This made me struggle in some aspects… When it comes to health, … you want to believe that it's not random. You want to believe that there's an intention, there’s a design, that there’s a plan. That, you know, if and when something bad happens, it’s not going to be for no reason… But can you accept that for yourself and for your family? You know, I think that is tough. (Dr 12)

I guess some days, there are just quiet nights or very early mornings when you just think about how life can be so transient, especially after losing a patient that you really fought quite hard for… You just kind of feel like life is really short. And it just makes me ponder about what happens after I die. I have no answer for that. (Dr 1)

#### Positive impact on relational ring

Some oncologists reported that their experiences with dying patients motivated them to appreciate and nurture relationships with family and loved ones. They also felt prepared to have conversations about care planning and death with their families, providing them with a sense of peace in knowing and respecting the wishes of their loved ones.

I want to spend more time with my parents…And I feel that that is something because being a physician makes me see that I do want to take care of my parents in their twilight years… I have these conversations with my family very early because of the experience that I’ve seen. And I've talked to them openly about their needs and their desires and what they want. I think that’s something that I'm very blessed to have because of my job and being put in such situations. (Dr 6)

Taking a leaf from all the patients I’ve seen, no one has ever said, ‘I should have worked harder.’ Almost everyone says that they miss the time with their children, with their family and everything. So, I guess that has influenced me in a way. (Dr 1)

#### Positive impact on individual ring

Working closely with cancer patients reaffirmed personal values and inspired a healthy shift in perspective for most oncologists. They reported a greater appreciation for “the little things,” adopted others-oriented thinking, and developed a newfound confidence in overcoming their insecurities.

It’s just that seeing patients, talking to them, them telling me what’s important to them, that really has got me thinking, essentially... It’s about what is important to me and how to cherish my days. (Dr 1)

For what was once a focus on what I wanted to accomplish became a focus on what more I can do for the people around me… how much I can actually do while remaining professional in my training, and at the same time, being true to who I am as an individual. (Dr 5)

### Domain 3: coping mechanisms

To face these challenges, the oncologists adopted several coping mechanisms. While most found ways to manage their emotions and balance work and personal life, a few developed maladaptive coping mechanisms and became more distant and detached.

#### Societal ring

To deal with the constant work demands and the increasingly hazy boundary between work and home, many oncologists accepted their limitations, and several learned to delegate aspects of their work to other specialists, including palliative medicine physicians.

In the past… I had to deal with all these issues myself. The nurse will say the family is very angry about what’s going on. These days, I just get palliative care…So, they rescue a lot of us by helping to sort things out. (Dr 5)

I’ve been to see my dying patient in a hospice before. It’s not something that I often do. I don’t do it to all my patients. There were some patients that I felt it was important for me to go and say my goodbyes to them … I’ve done it maybe only like two or three times. So, if you say, “Do you give 100%?” 100% means I should do it for all my patients, and that would burn me out. (Dr 5)

However, for some oncologists, the emotional toll of repeatedly witnessing death and dealing with difficult patients led to decreased empathy and detachment from patients.

Frustration is common because there are a lot of patients who make it difficult, in a way, to care for them, and sometimes I just don’t feel like making the effort to try to break that barrier down. There are some patients where it’s “You don’t want to, I’m fine. You don’t care for me, that’s fine. I don’t care.” (Dr 4)

It can be quite emotionally exhausting to get closer to all the patients. For me, I don't think I make a conscious effort to distance myself. But you know, I think it's very hard to say whether or not what the subconscious is doing. (Dr 10)

#### Relational ring

Oncologists often turned to their families for support but were also cognizant that this had its limits. Colleagues provided an effective support system for some oncologists due to their shared experiences. Through workplace and personal conversations, they were able to seek advice and express their grievances in safe environments.

I shared it with my husband. But you can’t share every case, and when you’re at home, there are other things to deal with. (Dr. 2)

Our colleagues are definitely very easy to share things with. They understand everything that is happening. Dr X has been my go-to when I was having this personal crisis. I do call him. The other person that I talk to is Z (a social worker). So intercollegial support, and ... seeking advice from those who have more experience in the field helps me to navigate and clarify certain thoughts and ideas. (Dr. 3)

#### Individual ring

Faced with distressing circumstances, many oncologists practiced reflection to help process their emotions, let go of situations beyond their control, and alleviate guilt and helplessness. Reflection also enabled the oncologists to prioritize their mental well-being and engage in self-care practices, including journaling, music, taking time off, and getting sufficient sleep.

It struck me because she was the first young patient that I really lost. So to me, immediately after the event, I was a bit sad that I couldn’t be there for her. But after a while, it came to the point wherein I just analyzed it objectively and said to myself, I did what I could. (Dr 4)

If I feel a very extreme emotion like anger or frustration, I have this Notes app on my phone and I have taken to just writing this long, long essay about what I’m frustrated with. Then, I just leave it there in the Notes app, and I never publish it or I never post it anywhere. But I just put it there and then it releases me in a way. (Dr. 4)

Relaxation, recreation, going home, watching some television, doing a bit of non-work things. I think that always helps. And… having a good sleep... These things usually help quite a lot. (Dr. 10)

#### Innate ring

To cope with the distress, some oncologists found solace in their religious or spiritual beliefs to make meaning of the suffering they witnessed.

It has showed me that God only gives you what you can handle. That statement has never rang more true to me than in previous years. It’s not that it made me closer to God. I just feel that God knows how I feel and how I am. And then, if I do my best to be a good person, God will have my back in a way. (Dr. 4)

I think religiously, I think with death, there is more to me … There is a lot more hope, there's a lot more promise, and there is the ultimate fulfillment and peace that life does not have at the moment. (Dr. 9)

## Discussion

In this qualitative study of oncologists, we found that caring for patients with cancer, particularly patients transitioning from curative management to palliative care has profound and enduring impacts on physicians’ beliefs, values, and attitudes — ultimately shaping their professional identity. The range of responses to patient illness and death highlights the myriad of sociocultural, personal, psycho-emotional, and professional factors at play, as framed by the rings of the RToP [39, 40]. These findings are consistent with studies involving palliative care nurses and physicians [10, 11].

The oncologists reported that the main precipitants for distress revolved around treatment decisions, notably the transition from curative treatment to palliative and end-of-life care. Trained to focus on curative treatment with the goal of prolonging life, the oncologists struggled with the decision to stop curative treatment, recognizing that ***“***once you stop, there’s no turning back.” This challenge went beyond justifying the decision to patients and families; it also involved defending their choices when confronted by other members of the healthcare team. Despite the team-based approach, oncologists often felt alone when making these life-changing decisions and experienced fear, guilt, and helplessness. This led them to question their life and career choices, seeking a better integration between dedication to patients and self-care and desiring improved work-life balance. Others faced existential threats as they confronted their mortality and fears of losing loved ones [11].

Another important finding from our study is that oncologists often do not feel well-equipped to handle the wider personal and psychosocial aspects of their role [41–48]. Several participants reported that their training focused on medical knowledge and clinical skills, with little focus on interprofessional teamwork [49–54], ethics [55–58], professionalism [23, 59–62], or communication skills [28, 63–65]. Recent reviews show that oncology training provides limited education and resources in self-care [29] or effectively dealing with compassion fatigue (CF), burnout, and moral distress. CF is described as “*a preventable state of holistic exhaustion that manifests in physical decline in energy and endurance, emotional decline in empathic ability, and spiritual decline as one feels hopelessness or helplessness to recover as a result of chronic exposure to others’ suffering *[66].*”* Rothenberger et al. describe burnout “*as the unintended net result of multiple, highly disruptive changes in society at large, the medical profession, and the healthcare system *[67].*”* Epstein et al. characterize moral distress as occurring “*when clinicians are constrained from taking what they believe to be ethically appropriate actions or are forced to take actions they believe are ethically inappropriate *[68]*.*” Curricular interventions should educate oncologists about complex psychosocial and cultural influences on care [69–82].

However, the study also illustrates how the professional identity of oncologists can be developed and supported through transitions of care. The physicians often used reflection to process their emotions, manage the loss of control in situations, and alleviate feelings of guilt and helplessness. This required the reframing of their role as physicians from one of providing a cure to one of providing comfort. Through this process, they made sense of their patients’ suffering and death by facilitating a “good death” for the patients and their families. These findings are consistent with prior studies of oncology doctors and nurses, where self-reflection was associated with decreased stress and burnout and helped to facilitate resilience [83]. Hospitals can implement programs that incorporate mindfulness and reflection to improve self-awareness and help physicians develop effective coping strategies. The oncologists in our study also turned to their spiritual beliefs to find meaning in life and death. Culture and religion can impact perceptions of death and dying [84]. Providing a safe space for all cultures and faiths can encourage reflection and introspection, enabling physicians to make meaning of their experiences.

Our findings also underscore the importance of enhancing social support for oncologists. The oncologists highlighted the importance of turning to their peers for support. Ho et al. [10] and Powell et al. [83] suggest that peer and organizational support help reduce moral distress, burnout, and compassion fatigue in healthcare professionals [83]. In studies of physicians and nurses, seeking and receiving support from colleagues, family, and friends after patient death helped lessen distress and improve emotional well-being [5, 83]. Interventions that facilitate real-time debriefing after patient death are effective in processing emotions and addressing unresolved clinical management concerns, bringing a sense of closure to the experience [16]. Multidisciplinary team debriefing models enable the oncologist to view the experience from different perspectives [85]. Physicians, however, may lack the confidence and skills to counsel their peers after a distressing patient care event [86]. Hospitals can offer peer-debriefing workshops that provide physicians with the knowledge and skills to offer timely support to their colleagues [86]. Other actionable support strategies include support groups, an on-call peer, and patient memorial services.

### Limitations

Our study has some limitations. The use of the RToP framework may limit the broader use of the findings of this small single-site study. Our results may also be specific to Singapore’s unique cultural landscape and healthcare system. Future studies are needed to further explore the impact of sociocultural considerations on personal principles and coping.

## Conclusion

Oncologists deal with feelings of guilt, helplessness, and self-doubt as they manage transitions in care. These experiences can have long-term effects on their personal and professional identity. The PIF of oncologists can be supported through self-awareness and reflection, peer support, and interventions to promote self-care, ultimately enabling them to make meaning of their experiences, renew family ties, and reaffirm their commitment to the profession. Our study highlights the need for hospitals to optimize support systems. This should include a review and re-engineering of the practice culture and environment to include longitudinal support consisting of mentorship or peer support [87], with effective training and professional development. Also important is access to guided reflective practice, debriefs, and access to well-being and self-care programs. These interventions should not be limited to oncologists but should also be open to the multidisciplinary teams that care for seriously ill patients and their families.

## Supplementary Information

Additional file 1. Interview Guide.

## Data Availability

The authors confirm that the data supporting the findings of this study are available within the article and its supplementary materials. To request data from this study, please contact Prof Lalit Kumar Radha Krishna (lalit.radha-krishna@liverpool.ac.uk).

## Abbreviations

PIF: Professional identity formation
RToP: Ring theory of personhood
SEBA: Systematic evidence-based approach
